# Diving Into the Diagnostic Score Algorithms of Heart Failure With Preserved Ejection Fraction

**DOI:** 10.3389/fcvm.2021.665424

**Published:** 2021-06-10

**Authors:** Dmitry Abramov, Purvi Parwani

**Affiliations:** Loma Linda University Health, Loma Linda, CA, United States

**Keywords:** heart failure, heart failure with a preserved ejection fraction, comorbidities, diagnosis, geriatics

Due to an aging population, heart failure with preserved ejection fraction (HFpEF) is on the rise. Yet this condition remains difficult to characterize and diagnose. There have been two recently proposed risk scores for the evaluation and diagnosis of patients with suspected HFpEF (1, 2). These include the European Society of Cardiology (ESC) consensus recommendation for the diagnosis of HFpEF (HFA-PEFF score) (1) and the H_2_FPEF (2) score. The H_2_FPEF score was developed from evaluation of patients with dyspnea and identified that obesity, hypertension, atrial fibrillation, pulmonary hypertension, older age (>60 years old), and evidence of elevated filling pressures on echocardiogram were associated with invasively confirmed elevation of filling pressures used as the gold standard for the HFpEF diagnosis. The HFA-PEFF score from the ESC is based on expert consensus and refers to a multi-step evaluation process of patients with dyspnea to diagnose HFpEF. The scoring systems aim to replace current simpler and phenomenological American College of Cardiology/American Heart Association definitions of HFpEF, which relies on signs and symptoms of heart failure, evidence of abnormal diastolic parameters, and preserved ejection fraction. This opinion piece offers concerns over attempts to protocolize a vastly heterogenous group of patients using diagnostic scoring systems.

## Co-Morbidities are the Rule

The HFA-PEFF algorithm suggests that evaluation of patients with dyspnea begin with ruling out cardiac and non-cardiac comorbid conditions that may mimic heart failure. Specifically, the algorithm targets coronary artery disease, lung disease, and anemia as comorbidities that need to be ruled out, but identifies obesity, diabetes, and atrial fibrillation as common risk factors in patients with HFpEF. However, teasing out the contribution of various comorbidities, including those that either mimic or are consistent with HFpEF, may be difficult in practice and have limited clinical implication (3).

The presence of one or more comorbid conditions like coronary artery disease, atrial fibrillation, hypertension, diabetes, renal insufficiency, pulmonary hypertension, anemia, obesity, and lung disease often defines older patients in the Western world. These comorbidities can be associated with fluid retention and dyspnea on exertion, which can mimic the signs and symptoms of heart failure. Many of these conditions, such as obesity, atrial fibrillation, systemic and pulmonary hypertension, and old age, have been specifically associated with elevated filling pressures at rest or with exercise as defined by the H_2_FPEF score (2). However, is there a need to label these comorbidities and their associated symptoms as garden variety HFpEF? Or, should the diagnosis and management of conditions associated with dyspnea and volume overload primarily focus on the comorbidities themselves?

These are key questions because calling the effects of these conditions HFpEF may distract caregivers from the management of the causal comorbidity. Indeed, older patients with multiple comorbidities are complex to evaluate and manage. The focus on the search for the HFpEF diagnosis may take the focus away from the in-depth evaluation, management, prevention, and discussion surrounding the comorbidities themselves. Medical care should always be directed at the true cause of illness, and treatment of co-morbidities has been suggested as the primary treatment of HFpEF (4). Patients with more severe manifestations of comorbidities may also have a worse prognosis, and comorbidities have been strongly associated with outcomes in patients with HFpEF (5). The prognostic implications of the HFpEF diagnosis, and the HFA-PEFF/H_2_FPEF scores (6), may therefore be due to comorbidity burden rather than a particular cardiac pathology.

## Integer scores/Invasive Evaluation for a Diagnosis of a Complex Syndrome

**Figure 1:**
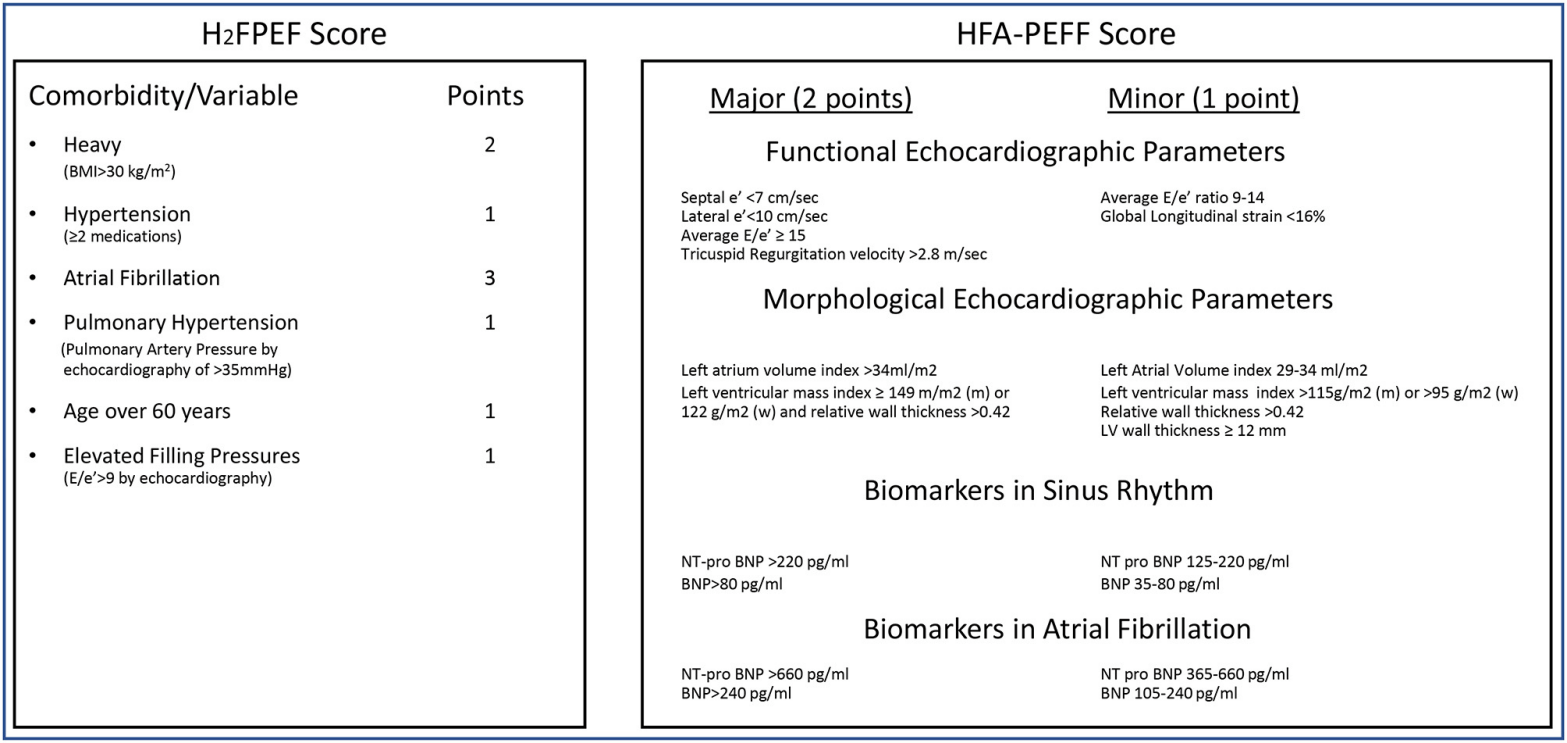
Scoring algorithms for HFpEF diagnosis. H_2_FPEF score: Patient gets points based on presence of comorbidity/variable. Low probability of HFpEF (0–1 points), Intermediate Probability of HFpEF (2–5 points), High probability of HFpEF (6–9 points). HFA-PEFF score: Each category is assessed, and patients get points if meeting a major or minor criteria. Intermediate score (2–4 points), High score consistent with HFpEF (≥ 5 points).

Both the HFA-PEFF and the H_2_FPEF algorithms rely on a scoring system to assess the likelihood of HFpEF (See Figure 1). While the H_2_FPEF score relies mostly on comorbidities, the HFA-PEFF scoring system is based on echocardiographic structural and functional parameters as well as natriuretic peptides. There are many challenges to the idea that an integer score, particularly as expressed in the HFA-PEFF algorithm, will help the care of complex patients.

First, the commonly used echocardiographic parameters for the diagnosis of HFpEF—diastolic abnormalities in mitral inflow and tissue Doppler as well as structural atrial enlargement or ventricular hypertrophy—have significant limitations as part of diagnostic algorithms (7) and echocardiographic subsets of HFpEF trials demonstrate a high number of patients with normal or only mildly abnormal diastolic/structural parameters. Despite these limitations, the HFA-PEFF score ultimately turns on echocardiographic structural and functional parameters with precise cut-offs to differentiate patients meeting normal, minor, and major criteria. However, strict precision in the measurement and interpretation of diastolic echocardiographic parameters may be difficult, which can complicate subsequent patient management. Based on the scoring system, many patients will score in the intermediate range, where the diagnostic algorithm becomes more complex and further evaluation with exercise diastolic stress testing or invasive hemodynamics at rest and/or with exercise is indicated (8).

Early experience with application of the diagnostic scores demonstrate significant discrepancy between the H_2_FPEF and the HFA-PEFF scores, with about a third or more of the patients with falling into the intermediate score range (6, 9). In this community, this may lead many older and frail patients who are being evaluated for HFpEF to be subjected to invasive or exercise testing as part of the guideline evaluation algorithms. Diastolic or invasive stress testing is not widely available in the community. There is also a lack of data on the feasibility, safety, efficacy, and cost effectiveness of advanced testing in the community for this common cohort of patients, and it is therefore unclear whether the benefits of pursuing complex testing outweigh the risks. Guidelines should reserve complex and invasive testing for tertiary care centers in patients who have atypical presentations, and the complex and invasive approach is unlikely to be either feasible or beneficial for most patients in the community who have dyspnea associated with multiple comorbidities and intermediate diagnostic scores. Additionally, many asymptomatic patients in an elderly cohort demonstrated intermediate or high-risk scores by the scoring systems (6), which may increase the risks of further diagnostic testing based on non-specific symptoms and scores alone.

## Rethinking the Label “HFpEF”

Finally, the application of the term “heart failure” to label this heterogenous syndrome deserves re-evaluation. As the doctor-patient relationship continues to evolve, there is an increased focus on optimizing communication to improve a shared understanding of illness. Part of this process may require the evolution of terms such as “heart failure” that may cause harm when interpreted by patients (10–12). The labeling of these findings as heart failure in clinical practice may lead to negative patient perception, especially since uncertainty exists about the underlying causal etiology of abnormal echocardiographic or lab findings which may not result from a “failing” heart.

**Figure 2:**
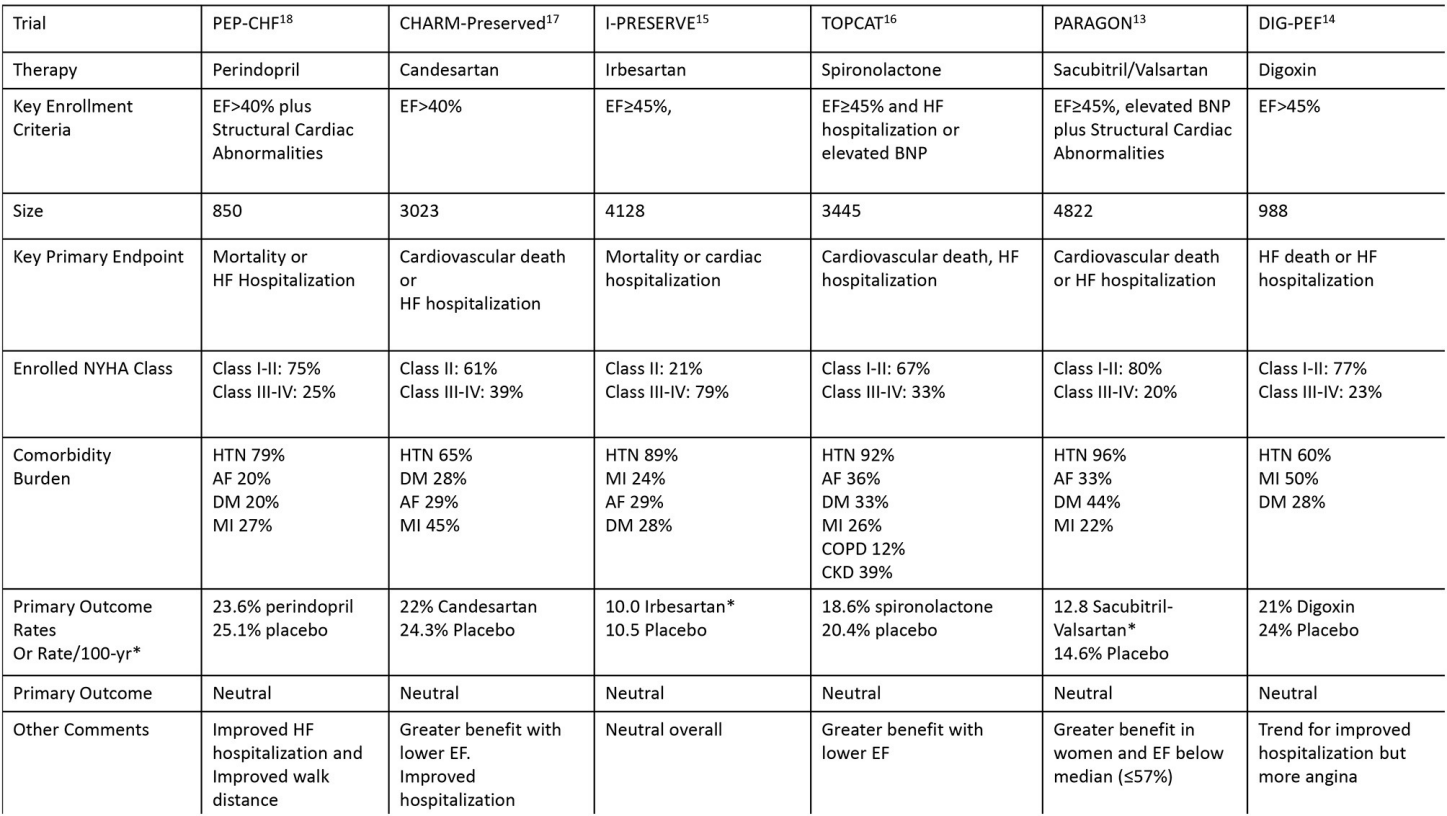
Key clinical trials involving patient with HFpEF.

The focus on optimizing terminology is particularly important because the diagnosis of HFpEF, regardless of the diagnostic algorithm, may not offer much change in management unless a specific comorbidity or disease process directly amenable to clinical management (i.e., cardiac amyloidosis) is identified. Importantly, a thorough evaluation of patient signs/symptoms (such as dyspnea or BNP elevation) and echocardiographic abnormalities (such as atrial enlargement or ventricular hypertrophy) can include specialist referral, ischemic evaluation, strain echocardiography, cardiac MRI, genetic testing, or other indicated testing which can lead to specific management decisions without the need to first establish a general HFpEF diagnosis. The multiple neutral clinical trials in patients with presumed HFpEF further suggest that the approach to diagnosis and management deserves re-evaluation (Figure 2) (13–18). The use of integer scores for clinical trial selection or quality metrics may face similar difficulties due to grouping of a heterogeneous cohort of patients.

## Conclusion

Much work is needed to optimize the diagnosis and management of a heterogeneous group of patients presenting for evaluation of dyspnea and volume overload. Future evaluation and management should focus on characterization of patient populations into subgroups based on underlying pathophysiology (19, 20). Under a targeted approach to diagnosis and management, patients with comorbidities such as diabetes, chronic kidney disease, or lab abnormalities such as BNP elevation, may be candidates for future clinical trials or novel medication classes without complex diagnostic evaluation. Likewise, patients with recurrent fluid overload manifested by hospitalizations for pulmonary edema may be candidates for implantable pressure monitoring systems without necessitating a search for a specific heart failure diagnosis. Evaluation and treatment targets based on atrial, microvascular, endothelial, and sympathetic nervous system dysfunction will continue to evolve, and these may lead to additional terminology and clinically meaningful diagnostic algorithms. In the meantime, diagnostic and management algorithms should be optimized with patients in mind, with less focus on heart failure terminology or dichotomous diagnostic cutoffs and more focus on understanding the pathophysiology of illness and obtaining management options that improve quality of life.

## Author Contributions

DA and PP conceived and wrote the manuscript. All authors contributed to the article and approved the submitted version.

## Conflict of Interest

The authors declare that the research was conducted in the absence of any commercial or financial relationships that could be construed as a potential conflict of interest.
